# Liposomes Affect Protein Release and Stability of ITA-Modified PLGA–PEG-PLGA Hydrogel Carriers for Controlled Drug Delivery

**DOI:** 10.1021/acs.biomac.3c00736

**Published:** 2023-12-22

**Authors:** Zuzana Kadlecová, Veronika Sevriugina, Klára Lysáková, Matěj Rychetský, Ivana Chamradová, Lucy Vojtová

**Affiliations:** † Central European Institute of Technology, Brno University of Technology, Purkyňova 656/123, 612 00 Brno, Czech Republic; ‡ Faculty of Chemistry, Brno University of Technology, Purkyňova 464, 612 00 Brno, Czech Republic

## Abstract

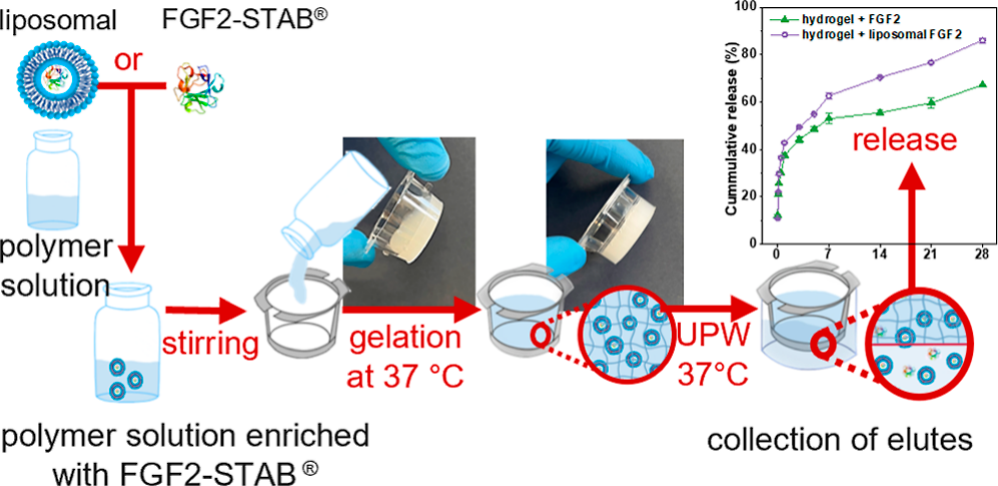

Fat grafting, a key regenerative medicine technique, often requires repeat procedures due to high-fat reabsorption and volume loss. Addressing this, a novel drug delivery system uniquely combines a thermosensitive, FDA-approved hydrogel (itaconic acid-modified PLGA-PEG-PLGA copolymer) with FGF2-STAB, a stable fibroblast growth factor 2 with a 21-day stability, far exceeding a few hours of wild-type FGF2’s stability. Additionally, the growth factor was encapsulated in “green” liposomes prepared via the Mozafari method, ensuring pH protection. The system, characterized by first-order FGF2-STAB release, employs green chemistry for biocompatibility, bioactivity, and eco-friendliness. The liposomes, with diameters of 85.73 ± 3.85 nm and 68.6 ± 2.2% encapsulation efficiency, allowed controlled FGF2-STAB release from the hydrogel compared to the unencapsulated FGF2-STAB. Yet, the protein compromised the carrier’s hydrolytic stability. Prior tests were conducted on model proteins human albumin (efficiency 80.8 ± 3.2%) and lysozyme (efficiency 81.0 ± 2.7%). This injectable thermosensitive system could advance reconstructive medicine and cosmetic procedures.

## Introduction

Free fat tissue has become surgeons’ best friend in reconstructive procedures. Autologous fat grafting (AFG) is common in regenerative medicine, and widely used to treat soft tissue defects, including breast augmentation.^1,2^ AFG’s main issue is the unpredictable volume loss after application due to the reabsorption rate, up to 40 to 60% in most patients.^3,4^ Without adequate integration, the graft fails to maintain its original volume and shape, leading to poor stability, more interventions, and prolonged recovery.^5,6^ Biocompatible hydrogels combined with lipoaspirate improve fat graft survival and ensure long-lasting filling volume.^7–9^ Using a “smart” thermosensitive injectable hydrogel carrier brings several advantages. The material is applied easily to the wound site and forms a gel with a defined stiffness in situ at human physiological temperature, supporting the structure of the injected wound area.^10^ Incorporation of therapeutic agents like growth factors is necessary for stimulating vascularization and new tissue granulation while slowly filling and replacing the AFG. The hydrogel reduces the fluctuation of active substances in vivo^11^ and supports their controlled release.^12–14^

A thermosensitive hydrogel based on poly(d,l-lactic acid-*co*-glycolic acid)-*b*-poly(ethylene glycol)-*b*-poly(d,l-lactic acid-*co*-glycolic acid) (PLGA-PEG-PLGA, also ABA) copolymer has been studied extensively^15,16^ and are approved by the Food and Drug Administration (FDA). Commercially available ABA-based hydrogel systems exist.^17^ ReGel is a hydrogel used for the controlled delivery of paclitaxel (OncoGel) in cancer treatment.^18,19^ However, ABA’s use as a drug delivery system is limited due to the low degree of functionality. In this study, the copolymer’s end structure is modified with itaconic acid (ITA), resulting in the α,ω-itaconyl (PLGA-PEG-PLGA) (or ABA-ITA), according to the method published by our group.^20^ ITA is derived from renewable resources,^21^ can be catabolized in mammalian liver mitochondria,^22,23^ and is also FDA-approved. End-modification introduces double bonds and carboxylic groups on both ends of the copolymer chain,^20^ resulting in mucoadhesion.^24,25^ Rheological properties and thermoresponsive behavior of ABA-ITA copolymer have been published.^10^ The copolymer chain is amphiphilic; the hydrophilic PEG block is between two hydrophobic PLGA blocks. The copolymer self-assembles into micelles above the critical micellar concentration forming an elastic gel in the aqueous environment.^26–28^

Adding pro-healing bioactives to the hydrogel carrier can accelerate vascularization at the wound site.^29,30^ In this study, a thermostable variant of fibroblast growth factor-2 (FGF2), FGF2-STAB is used. FGF2 stimulates vascularization and supports tissue granulation and wound healing.^31,32^ FGF2-STAB retains its total biological activity even after 20 days at 37 °C.^33,34^ Our group’s previous study^35^ was focused on the incorporation of FGF2-STAB into the mucoadhesive ABA-ITA hydrogel gradually releasing the protein over 21 days at a pH below 6. The modified hydrogel degrades differently since the dissociation of the carboxyl groups in the ITA leads to rapid degradation and lower pH levels.^28^ Therefore, in this study, the FGF2-STAB protein, in comparison with two model proteins, namely, human lysozyme and human serum albumin, were encapsulated into liposomes to be protected against a low pH environment during the polymer degradation as decreasing the pH might result in protein unfolding.^36^ Protein release, hydrogel decomposition, and changes in rheological properties with liposome addition are monitored for potential drug delivery at the wound site.

## Experimental Section

### Materials and Methods

#### Materials

The chemicals for **copolymer** preparation, d,l-lactic acid (d, l-LA) ≥ 99.5% and glycolic acid (GA) with ≥99.9% purity, were purchased from Polysciences (USA), poly(ethylene glycol) (PEG) with Mw 1500 g·mol^–1^ was purchased from Merck (Germany), and the tin catalyst Sn(II) 2-ethyl hexanoate ≥92.5% was purchased from Sigma-Aldrich (USA). The itaconic anhydrite 98% was purchased from Acros Organics, Thermo Fisher Scientific (Czech Republic). For copolymer characterization, deuterated chloroform (DCl3, Merck, USA) and tetrahydrofuran (THF for high-performance liquid chromatography—HPLC, ≥99.9%—Merck, USA) were used. For **liposome** preparation, the 1,2-dipalmitoyl-*sn*-glycerol-3-phosphocholine (DPPC) 16:0 with purity >99% was purchased from Avanti Polar Lipids (USA). Analytical-grade glycerol was purchased from Sigma-Aldrich (Germany). Ultrapure water (UPW) Type 1 (ISO 3696) was prepared using the Millipore purification system Milli-Q Academic (France). The FGF2-STAB was kindly provided by Enantis, L.t.d. (Czech Republic); human lysozyme was purchased from Merck (USA), and human albumin was purchased from Sigma-Aldrich (USA). For the analysis of released proteins, the Bradford reagent for 0.1–1.4 mg·ml^–1^ protein was purchased from Merck (USA).

### Liposome Preparation

Liposomes were prepared following the principles of the Mozafari method.^37,38^ The DPPC (2 wt %), UPW, and glycerol (3% v/v) were placed in a round-bottom flask connected to a protective nitrogen atmosphere. The flask was then placed in an oil bath, preheated at 60 ± 1 °C, and the mixture was stirred using a hot plate stirrer (IKA, Germany) at 1200 rpm for 1 h. The liposomal mixture was then sonicated using a laboratory bath sonicator (Ultrazvuk, Czech Republic). The water in the bath sonicator was heated to 61 ± 1 °C, and the sample was sonicated for 2 × 20 min. The sample appearance changes from turbid to translucent showed the correct sonication process. For empty liposomes, the mixture was transferred into a glass vial, and the annealing process was held in a water bath, preheated at 43 ± 1 °C and the liposomal solution was mixed at 400 rpm using the hot plate stirrer (IKA, Germany) for 1 h. Afterward, the liposomal mixture was left at ambient temperature for 1 h. For protein-encapsulated liposomes, the amount of drug was weighed into the glass vial, and then, the liposomal mixture cooled to 43 ± 1 °C was introduced. The concentration of proteins (FGF2-STAB, human Lysozyme, and human serum Albumin) in each formula was 500 μg·ml^–1^.

### Liposome Characterization

The hydrodynamic diameter (*d*_h_), polydispersity index (PDI), and zeta potential (ZP) were determined in all formulations within 24 h after the preparation. The ZP was determined using the ZetaSizer Nano ZS (Malvern Instruments, UK) at 25 °C with the 633 nm laser using the disposable folded capillary zeta cells DTS1070. The diameter and PDI were determined using the dynamic light scattering (DLS) detector (Wyatt Technology, USA) operating with a 658 nm laser at 25 °C and a 90° detector angle. Measurements were done using the Wyatt Technology single-use DLS cuvettes containing 5 μL of diluted (0.1% v/v) liposomes. ZP and DLS measurements were performed in six measurements per sample. The morphology was observed using the scanning electron microscope MIRA3Raith (TESCAN, Czech Republic) in a scanning transmission (STEM) mode. A drop (∼15 μL) of diluted liposome solution was placed on carbon-coated 200 Mesh copper grids (Agar Scientific L.t.d., Stansted, UK) and left overnight to dry. The grids were viewed under STEM at suitable magnifications at an acceleration voltage of 20 kV. The encapsulation efficiency (EE) was determined as follows, 1 mL of the liposome suspension was pipetted into an Eppendorf tube, and the liposomes were separated from the unencapsulated proteins by centrifugation at 25 °C (13,500 rpm, 15 min) using the Micro Star 12 microcentrifuge (VWR, Czech Republic). The supernatant was carefully collected and subjugated to Bradford protein assay. To determine the protein released from the liposomes, 1 mL of UPW was poured over the sedimented liposomes, agitated, and placed into a thermostat at 37 °C. This process lasted 28 days, and supernatants were collected after 1, 5, and 12 h and then after 1, 5, 7, 14, 21, and 28 days.

### Synthesis, Modification, and Purification of ABA-ITA Copolymer

The hydrogel-based ABA-ITA was used as a liposome and protein carrier system. The polymer with the PLGA/PEG weight ratio of 2.5 and the d, l-LA/GA molar ratio of 3.0 was synthesized by ring-opening polymerization (ROP) on a Schlenk’s line using a tin catalyst under a nitrogen atmosphere at 130 °C for 3 h. The ABA modification was subsequently performed in bulk by itaconic anhydride (2.5 molar ratio to polymer) under the nitrogen atmosphere for 1 h at 110 °C. The crude ABA-ITA was purified three times from the soluble low-molecular-weight polymers and unreacted monomers by dissolving in ultrapure water (pH 6.7) and then precipitated at 80 °C. The purified copolymer was separated by decantation, freeze-dried until the constant weight, and stored in a fridge.^20^

### Characterization of ABA-ITA and Hydrogel Preparation

The copolymer molecular weight, PLGA/PEG ratio, and LA/GA ratio were characterized by using proton nuclear magnetic resonance ^1^H NMR spectroscopy (60 MHz, Spinsolve 60, MAGRITEC, Germany). The copolymer was dissolved in DCl_3_ with a concentration of 20% w/v. 128 scans were used for each sample, and the measurements were performed at 25 °C. ^1^H NMR spectra were recorded by using an ACD/1D NMR Processor. Gel permeation chromatography (GPC)/size exclusion chromatography (SEC) with a multiangle light scattering detector (MALS, DAWN HELIOS-II, Wyatt, USA) and refractometer (T-rEX, Wyatt, USA) was used for the number of average molecular weight (*M̅*_n_) and the polydispersity index (*M̅*_w_*M̅*/_n_) detection. Two columns (Plgel 5 μm Mixed-C) were used for separation, and THF with a flow rate of 1 mL·min^–1^ was used as the mobile phase.

The polymer was dissolved in UPW and stirred for 4 days at 12 °C to obtain the hydrogel with a polymer concentration of 20% w/w. The hydrogel was then subjected to dynamic rheological analysis to determine the hydrogel gelation properties on an advanced rotational rheometer DHR2 (TA Instruments, USA). The temperature ramp test was carried out from 25 to 55 °C at a heating rate of 0.5 °C·min^–1^, constant angular frequency of 1 rad·s^–1^, 1% strain, and gap 700 μm (plate–plate geometry, 20 mm Standard Peltier). Moreover, a solvent trap was used to prevent solvent evaporation during the experiment.

### ABA-ITA Hydrogel Enrichment and Degradation

Afterwards, the nonencapsulated and liposome-encapsulated proteins were added to the prepared hydrogel so that the final concentration was 100 μg·ml^–1^ in each ABA-ITA hydrogel scaffold. The concentration of empty liposomes was not directly calculated. Instead, the volume aliquot of empty liposomes was added to match the volume of the unencapsulated (or liposome-encapsulated) protein solution. The mixture was stirred at 12 °C for 30 min and transferred to the cultivation plate insert (SPL InsertTM Hanging, 6 inserts/6 well plate, PC, 0.4 μm, Thermo Fisher Scientific, USA). The gelation at 37 °C for 45 min followed, and the insets were submerged into a UPW environment in a bottom pan of the well plate. The samples were left in an incubator at 37 °C. As the hydrogel degraded through the 0.4 μm polycarbonate membrane into the UPW or the PBS, the insets containing undegraded hydrogel were weighted, and the elute in the bottom pan was collected. The bottom pan was then refilled with the same volume of fresh UPW, and the inset was submerged back in to continue the degradation process. The whole degradation process lasted 28 days. Elutes were collected 1, 3, 5, and 12 h after 1, 3, 5, 7, 14, 21, and 28 days. The mass change and pH levels of the collected elute (pH meter H138 miniLab, Hach, USA) were determined, and the proteins presented in the elutes were subjugated to the Bradford protein assay.

### Bradford Protein Assay

Ultraviolet–visible (UV–vis) light spectrophotometer (Biochrom Libra S22, UK) was used to measure the collected elutes and supernatants. The assay was performed using the Bradford reagent at 595 nm. Three calibration curves were used in a concentration range of 1–10, 10–100, and 100–1000 mg·ml^–1^ to determine the protein concentration. The pipetting ratios of the sample and Bradford reagent quantities are shown in Table 1. Each sample was measured in a technical duplicate.

**Table 1:** Bradford Reagent/Sample Pipet Volume for a Single Measurement for Each Calibration Depending on the Expected Concentration of Protein in the Sample

calibration	concentration (μg·ml^–^^1^)	reagent (μL)	sample (μL)
low	1–10	500	500
middle	10–100	800	200
high	100–1000	1000	30

The encapsulation efficiency was calculated as follows
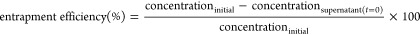


The concentration in the supernatants collected from the liposomes was also calculated according to the calibration curves. The initial concentration was considered equal to the concentration effectively entrapped in the liposomes.

### Statistical Analysis

The data were statistically evaluated using OriginPro 2020b, and the results were presented in the form of text accompanied by plots and graphs. Data normality was analyzed via the Shapiro–Wilk test. Results are expressed as mean ± standard deviations. Multiple comparisons of means (Tukey test) were used to evaluate statistical differences between groups. For non-normally distributed data, the Kruskal–Wallis ANOVA was used. Principal component analysis (PCA) was used to visualize the multivariate data. The *p*-values < 0.05 were considered to indicate statistically significant results.

## Results and Discussion

Liposomes prepared by the Mozafari method are an attractive “green” alternative to conventional methods since this method does not include organic solvents or high temperatures and therefore is ideal for encapsulating heat-sensitive active substances, including enzymes or proteins forming nontoxic liposomal carriers.^39^

### Liposome Characterization and Encapsulation Efficiency

**Figure 1:**
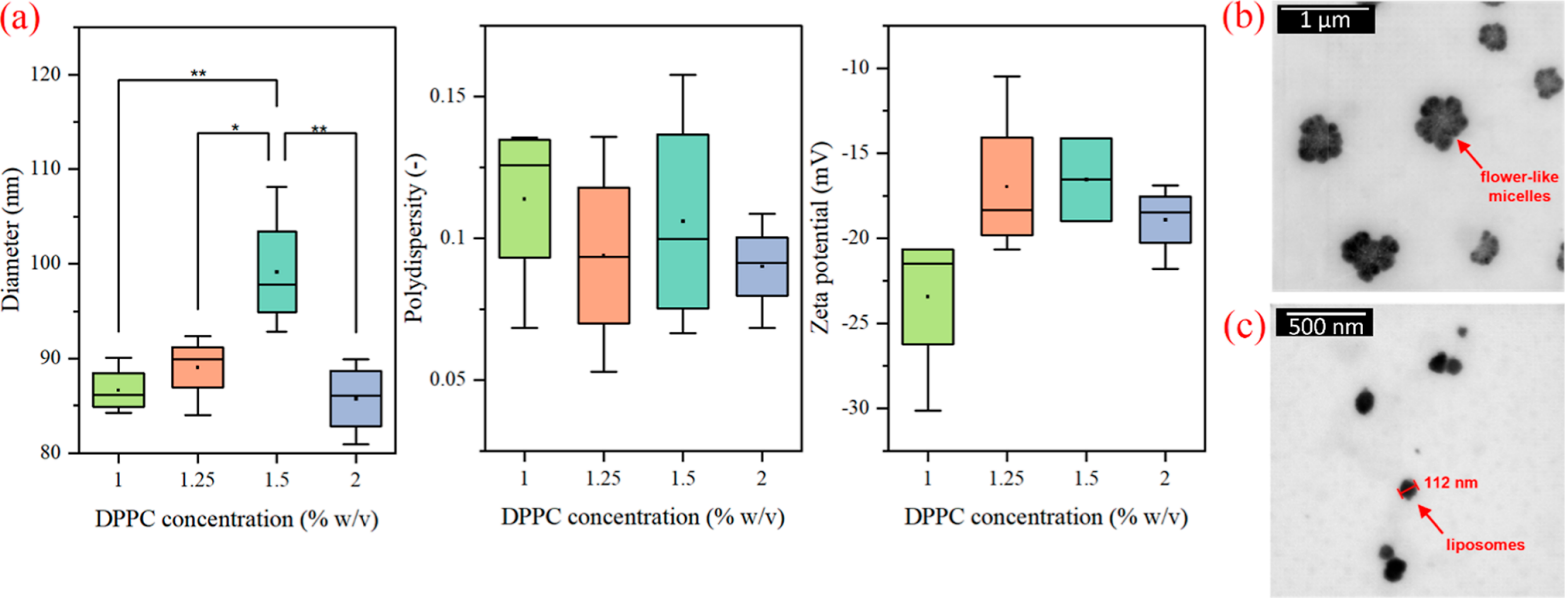
(a) Diameter, PDI, and ZP of formulations containing 1.0, 1.25, 1.5, and 2.0% w/v of DPPC, STEM micrographs showing the apparent difference between the flower-like micellar morphology of low DPPC concentration formulations such as 1.0% w/v (b) and a spherical liposomal morphology on higher DPPC formulations containing 2.0% w/v DPPC (c). *P* values resulting from the Tukey’s test reaching statistical significance (*p* < 0.05) were marked *; *p* values reaching statistical significance (*p* < 0.01) were marked **.

Liposomes with different weight concentrations of DPPC were prepared at first, namely, 1.0, 1.25, 1.5, and 2.0% w/v (*n* = *4*). The diameter, PDI, and ZP of each formulation are displayed in Figure 1a. The formulations gave a negative ZP in the range consistent with the measurements of Mosharraf et al. on DPPC liposomes,^40^ with PDI <0.35, indicating acceptable homogeneity.^41^ Liposomes exhibited an average particle size in the range of 85.7 to 99.2 nm, giving a particle size comparable to liposomes prepared by conventional methods,^42^ which has not been shown yet on the Mozafari method-prepared liposomes.^43–46^

The results show that the concentrations of 1.5 and 1.25% (w/v) give the least satisfactory results, as the liposomes are relatively large with a wide PDI range and high ZP. Specifically, liposomes with a concentration of 1.25% (w/v), despite having a diameter of around 90 nm, exhibited a broad polydispersity range with significant variations between the measurements and the prepared samples. Similar inconsistencies were observed in the ZP measurements, with the ZP values being notably higher, even falling below −20 mV. These findings strongly indicated that a formulation with this concentration yielded irreproducible results. The concentration of 1.5% w/v was not chosen for the application due to its larger diameter compared to other prepared formulations, all of which exhibited a wide range of sizes/diameters as Figure 1 shows. Similarly, the 1.25% w/v formulation showed a wide polydispersity range along with a low ZP. Since ZP has a direct impact on the overall stability of the liposome system, the objective was to select the formulation with the lowest ZP. The lowest ZP was observed in the 1.0% w/v formulation. However, the morphology of this formulation did not match that of liposomes, compared to the morphology of the 2.0% w/v formulation in Figure 1b. The 1.0% w/v formulation gave more of a “flower-like-micelle” morphology, as shown in Figure 1c.

The concentration of 2.0% w/v was selected as an ideal candidate since the formulation gives small liposomes (85.73 ± 3.85 nm) with a narrow PDI (0.09 ± 0.02) and a ZP of −18.91 ± 2.08 mV, adequate for colloidal stability, together with the STEM images confirming a spherical liposomal morphology.

**Figure 2:**
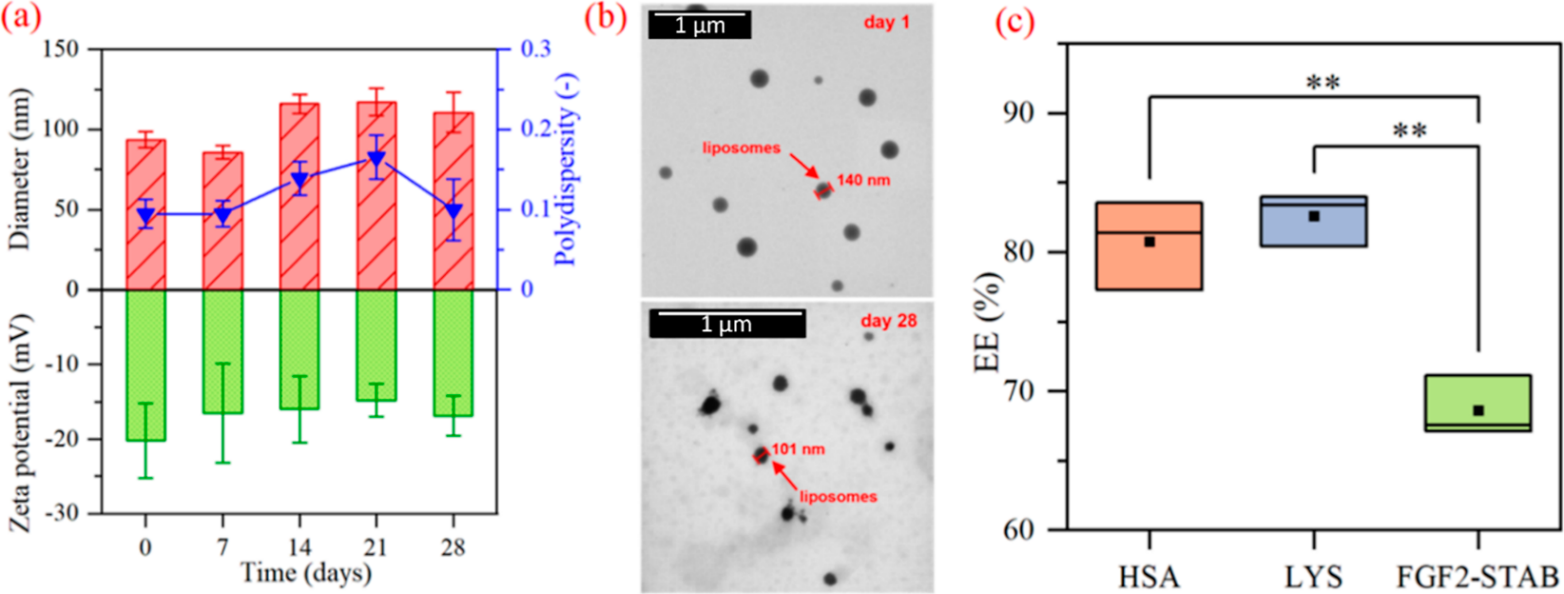
(a) Diameter, PDI, and ZP characteristics of 2.0% w/v liposomes over 28 days. The STEM micrographs of 2.0% w/v formulations (b) show no significant change in morphology between day 1 (top) and day 28 (bottom). The paired comparison of means on EE of HSA, LYS, and FGF2-STAB by Tukey’s test is shown in (c). *P* values reaching statistical significance (p < 0.01) were marked **. ABA-ITA copolymer characterization.

Nevertheless, the main issue with liposome formulations, in general, is the system’s long-term stability, contributing to the release effectiveness in the intended application. Several studies have shown that formulas prepared with glycerol were stable over longer periods^47,48^ and we have confirmed these findings as the prepared formulas showed adequate storage stability regarding ZP when stored at 25 °C (*n* = 5). The change in liposomal properties is shown in Figure 2a, and the change in morphology is shown in Figure 2b. As the diameter and PDI enlarged with time, the ZP decreased. Poudel et al.^49^ studied the stability of Mozafari-method-prepared liposomes at 4 °C. The change in liposome diameter and the enlargement of deviations match the results obtained in our experiment.

The encapsulation efficiency (EE) was tested on FGF2-STAB and two model proteins, namely, human lysozyme (LYS) and human serum albumin (HSA). LYS and FGF2-STAB have a slightly smaller molecular weight^50^ (compared to HSA having a larger molecular weight^51^). The data show that there is a significant statistical difference (*p* < 0.01) between these three proteins, shown in Figure 2c. The FGF2-STAB gave much smaller EE (68.6 ± 2.2%) compared to HSA (80.8 ± 3.2%) and LYS (81.0 ± 2.7%).

The surface of HSA has 11 hydrophobic binding sites^52^ and therefore was expected to give slightly lower EE.^53^ Al-Ayed et al.^54^ have shown that HSA significantly alters the physical state of the liposome membrane. That might result in the incorporation of HSA into the liposomal bilayer membrane. On the contrary, LYS has more surface polar groups^55^ and, therefore, would encapsulate within the liposome’s core giving EE over 80%, as Lopes et al.^56^ have shown in their experiments. The FGF2-STAB was expected to give similar results since the protein is more hydrophilic due to the Arg, Cys, and Ser residues on its surface.^32^ The measured EE was significantly lower. However, this might have been caused by a relatively small ratio of drug/lipids. In our study, the ratio of 1:90 was applied to be the most effective on model proteins HSA and LYS, but a higher ratio of 1:300 could enhance the EE up to 90%, as proved by Xu et al.^42,57^ To facilitate a fair comparison of the encapsulation efficiency across various proteins, it was essential to maintain uniform protein concentrations in all observed formulations; therefore, the ratio of 1:90 was used for all experiments.

The ABA-ITA copolymer average molecular weight and PDI were determined using GPC analysis, since one molecular weight value cannot be established for polymers. Molecular weights, LA/GA molar ratios, PLGA/PEG weight ratios, and amount of end-capped ITA were measured by ^1^H NMR spectroscopy. Molecular weights and chemical composition were similar to theoretical values.^58,59^ All copolymer characteristics are described in Table 2.

**Table 2:** Average Values of the ABA-ITA Measured on GPC and NMR, Compared with the Theoretical Values

		ABA-ITA (theoretical)	ABA-ITA (*n* = 6)
*M*_n_ (g mol^–^^1^)	GPC	5250	6090 ± 300
	^1^H NMR		5290 ± 130
	PDI		1.13 ± 0.04
PLGA/PEG (w/w)		2.5	2.53 ± 0.09
LA/GA (mol/mol)		3.0	3.03 ± 0.25
ITA (mol %)			74.6 ± 8.6

### Structural Stability of the ABA-ITA Hydrogel Scaffold

**Figure 3:**
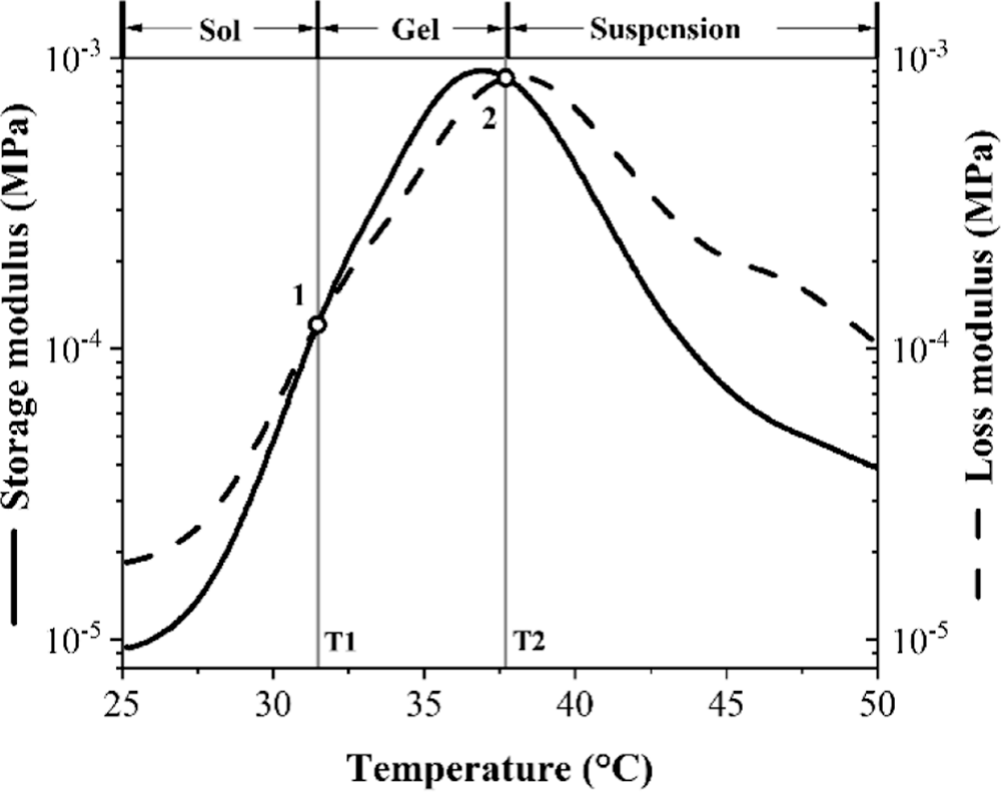
Characteristic temperatures of the ABA-ITA hydrogel at a heating rate of 0.5 °C/min.

The structural stability of the ABA-ITA hydrogel at different temperatures and the sol–gel and gel–sol transitions was indicated using rheological analysis. The transition temperatures were defined by the relationship between the storage (*G*′) and loss (*G*″) moduli, schematically illustrated in Figure 3 on the pure ABA-ITA hydrogel. At low temperatures, both *G*′ and *G*″ increase along with increasing temperature, and *G*′ gradually becomes larger than *G*″, implying that the copolymer solution becomes stiffer. Here, the gelation temperature (T1, sol–gel phase transition) is defined at the cross-point of *G*′ = *G*″ (point 1). The second intersection of *G*′ and *G*″ (point 2) specifies the beginning of decay of the gel structure (T2, gel–sol phase transition). Additionally, there is third phase–suspension, with a small contribution of elastic properties. This suspension phase is not important for our further examination.

**Figure 4:**
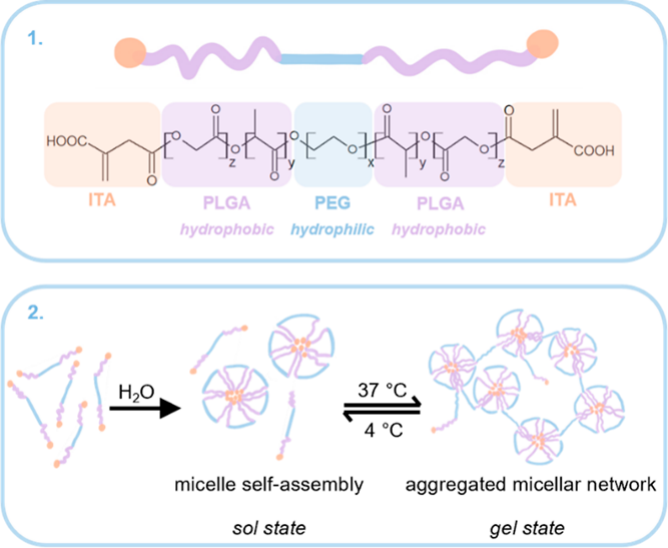
Α,ω-itaconyl (PLGA-PEG-PLGA) polymer chain (1), the self-assembly of the amphiphilic copolymer structure in aqueous solution into micelles, followed by the formation of a micellar network at temperatures above the upper critical solution temperature (2).

The rheological data of a 20% w/w solution of the ABA-ITA show that the sol–gel transition (T1) occurs at a temperature of 31.50 ± 0.31 °C (see Table 3). The second transition T2, the collapse of the gel structure, appears at 37.83 ± 0.19 °C. This confirms the thermosensitivity of the ABA-ITA copolymer and its ability to gel under physiological conditions at a given concentration. Based on the results, the presence of HSA, LYS, and FGF2-STAB slightly shifts the gelation point to higher temperatures, within 1 °C. This implies that protein addition affects the thermodynamics of the micelles which are the base of the ABA-ITA hydrogel structure (as shown in Figure 4) to a certain level.

**Table 3:** Transition Temperatures of Analyzed Samples, LIP(*x*) Indicates the Addition of Liposome-Encapsulated Protein

sample	T1,°C	T2,°C	sample	T1,°C	T2,°C
ABA-ITA	31.50 ± 0.31	37.83 ± 0.19	ABA-ITA + LIP(none)	31.49 ± 0.26	38.10 ± 0.27
ABA-ITA + HSA	32.37 ± 0.07	37.75 ± 0.04	ABA-ITA + LIP(HSA)	32.57 ± 0.02	38.08 ± 0.04
ABA-ITA + LYS	32.47 ± 0.30	38.01 ± 0.11	ABA-ITA + LIP(LYS)	31.98 ± 0.30	38.18 ± 0.05
ABA-ITA + FGF2-STAB	32.36 ± 0.32	38.43 ± 1.21	ABA-ITA + LIP(FGF2-STAB)	30.10 ± 1.03	37.35 + 0.07

By contrast, the liposomal nanoparticles (LIP) barely change the sol–gel transition temperature (31.49 ± 0.26 °C). Although liposome-encapsulated HSA, LIP(HSA), and LYS, LIP(LYS) slightly move the gelation point to 32.57 ± 0.02 and 31.98 ± 0.30 °C, respectively. On the contrary, liposome-encapsulated FGF2-STAB, LIP(FGF2-STAB) moves the gelation point to 30.10 ± 1.03. Therefore, enrichment with proteins or liposomes does not drastically impact the hydrogel’s ability to form a gel in the aqueous medium at physiological temperature. Compared with the proteins that are not encapsulated, the interaction of the proteins with the copolymer chain and its subsequent impact on micellar gelation cannot be observed in the case of liposome-entrapped proteins.

Rheological properties of the itaconic acid modified PLGA-PEG-PLGA copolymer were extensively studied by our group^10,35^ at a body temperature of 37 °C, which is the temperature that experiments of this study were conducted at as well. When we performed temperature-sweep measurements on the protein-enriched and liposome-enriched hydrogel formulations, we did not observe any shift in the characteristic sol–gel and gel–sol phase transition temperatures. This lack of change in temperature behavior led us to anticipate that there would be no significant alterations in the rheological properties. Consequently, we made a general assumption that the hydrogels have retained their thixotropic behavior and their mechanical properties.

### Hydrolytic Stability of the ABA-ITA Hydrogel Scaffold

**Figure 5:**
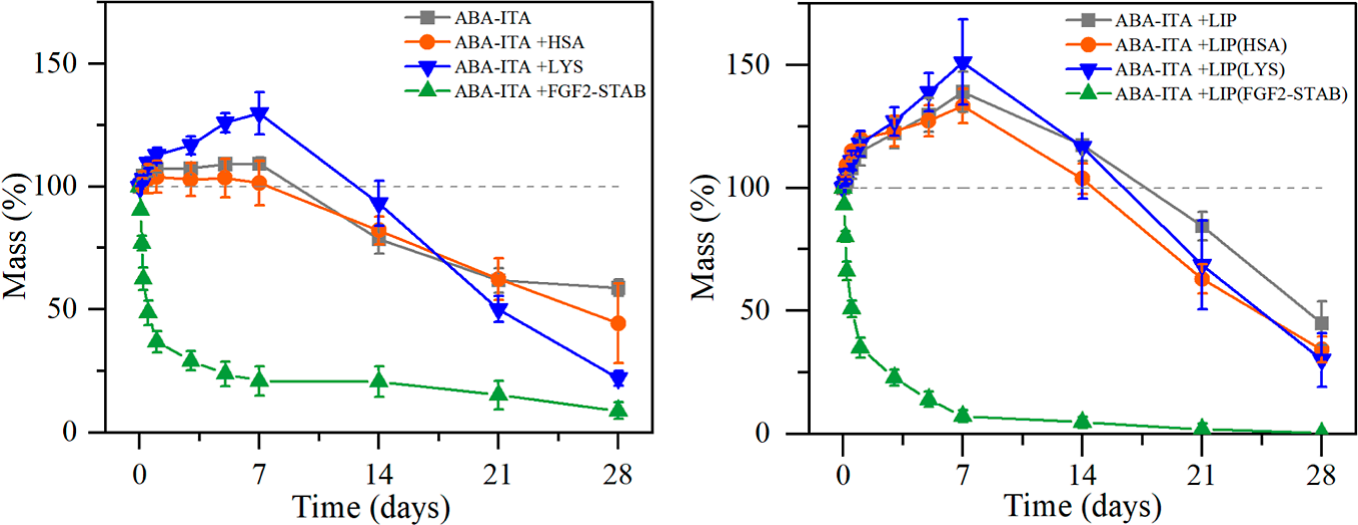
Mass loss of ABA-ITA 20% w/w hydrogel in the UPW over 28 days, on the left, the influence of simple proteins and on the right the influence of empty liposomes and liposome-encapsulated proteins.

The mass change of the ABA-ITA hydrogel in water (*n* = *5*) is shown in Figure 5. Pure hydrogel, hydrogel enriched with HSA, and LYS showed similar degradation curves. The hydrogel absorbs water and swells for the first 7 days as the swelling reaches around 4% in ABA-ITA, 10% in HSA, and up to 30% in LYS-loaded hydrogel. This triggered the hydrolysis of ester bonds present in the copolymer structure, and thus, after 7 days, the structure starts to collapse, leading to a gradual decrease in mass until 53.78 ± 2.02% of the original hydrogel was still present. The hydrogel erosion is much more evident in the LYS-enriched hydrogel carrier, where only 22.09 ± 2.90% of the original mass resided. However, the presence of FGF2-STAB results in almost exponential mass loss; after 7 days, only 21.02 ± 5.89% resided. As our group reported^58^ on the ABA-ITA stability, the carboxylic end groups on the ITA are hydrated first. A part of the ester bonds follows, leading to the decrease in the aggregated micellar network and pore formation, in which large amounts of water can be absorbed, as shown in the mass change of ABA-ITA in the left part of Figure 5.

Our previous study^35^ reported on the difference between the polarity and molecular weights of the HSA and LYS, which tends to bind to the surface of the ABA-ITA micelle, and the only part of it is hidden in the centre, while a more hydrophobic HSA is bound to the centre of the micelle (the micellar formation is shown on Figure 4).

In our study, both proteins impacted the stability of the aggregated micellar network of the copolymer. However, the situation with FGF2-STAB was somewhat different. The results indicate that the net positive charge on FGF2-STAB in physiological conditions (isoelectric point 9.6^60^) might lead to the binding of the amine groups on the protein to the carboxyl group on the ITA in the polymer chain via ion interactions. This would explain the faster hydration of the carboxylic groups on the ITA and ester bonds present in the PLGA part of the chain. A similar change in mass is also observed on the liposome-encapsulated FGF2-STAB, indicating that the same phenomena happened. Even though the FGF2-STAB is encapsulated in the liposomes, the burst release of the protein was determined within the first day of the measurement, and therefore, the concentration of the protein might be sufficient to hinder the micellar gelation and lower the hydrolytic stability of the system. These results correspond to a lower encapsulation efficiency of FGF2-STAB (EE of 68.6 ± 2.2%) compared to HSA (80.8 ± 3.2%) and LYS (81.0 ± 2.7%), as the “free” FGF2-STAB present in non-negligible amounts from the beginning of the degradation study.

**Figure 6:**
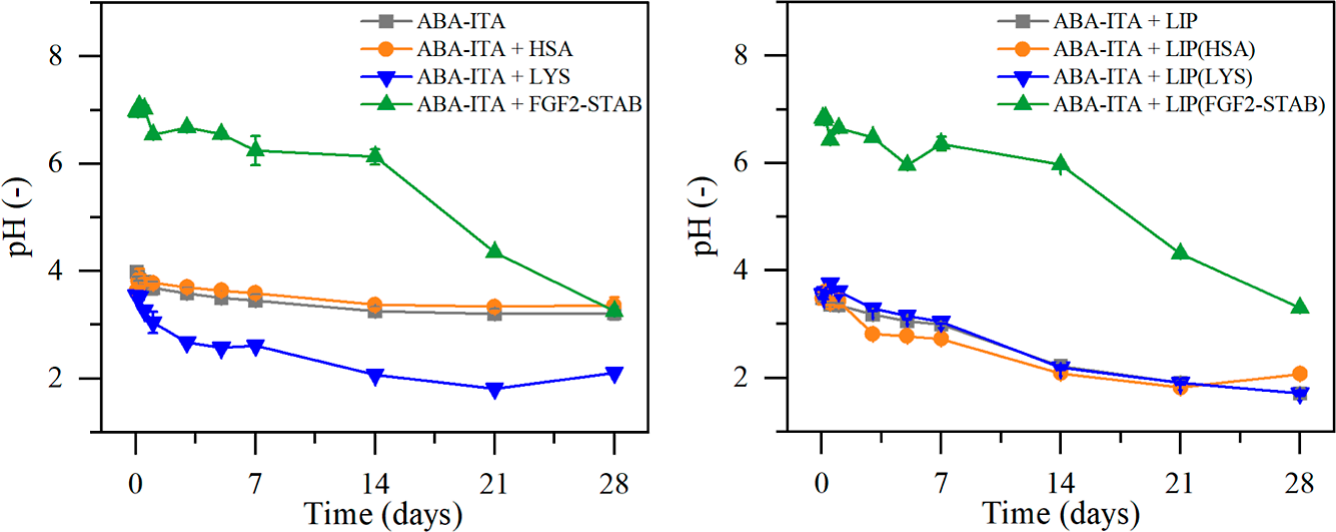
Change in pH levels in the hydrogel elutes over the 28 day period. On the left, the influence of the addition of simple proteins compared to unenriched hydrogel; on the right, the influence of liposome-encapsulated proteins compared to empty liposome enriched hydrogel.

The pH levels were measured on elutes from each sample, shown in Figure 6. The pH levels decreased gradually in hydrogels without liposomes, from 3.72 ± 0.04 on the first day to 2.89 ± 0.68 on day 28. In hydrogels with liposomes, the pH changed from 3.51 ± 0.04 on the first day to 1.83 ± 0.21 on day 28. The very low pH was measured on all of the liposome-enriched hydrogels compared to hydrogels without liposomes. The difference in pH levels might have been caused by the charge on the liposomes, supporting the hydrolysis of the ester bonds in the ABA-ITA structure and leading to a faster production of acidic degradation products (LA and GA), which would result in lower pH levels in the elutes.

### Protein Release

**Figure 7:**
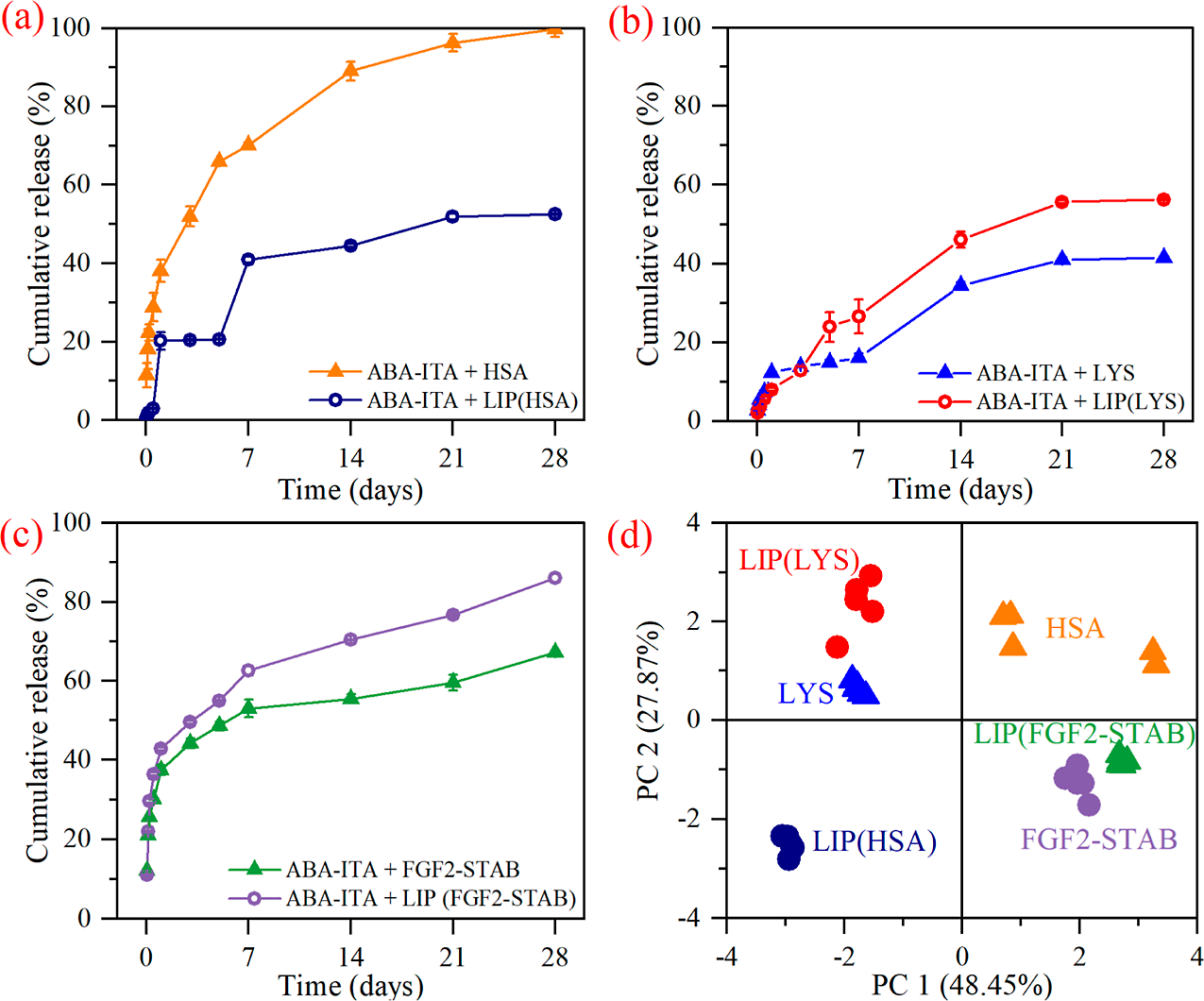
Release of HSA (a), LYS (b), and FGF2-STAB (c) from ABA-ITA 20% w/w hydrogel and the PCA (d).

The release mechanism was observed for each protein separately (*n* = 5). Two systems were compared, ABA-ITA enriched with a simple protein (ABA-ITA + HSA, ABA-ITA + LYS, ABA-ITA + FGF2) and ABA-ITA enriched with an encapsulated protein [ABA-ITA + LIP(*x*)], as shown in Figure 7. The resulting release curves show that the ITA-modified matrix gives quite different release mechanisms than the previously studied unmodified ABA matrix.^35^ Because the protein release process is influenced by several factors that correlate with each other (correlation factors >0.3), PCA was used to streamline the interpretation. The two principal components used in this case together carry 76.32% of the original variability. Component 1, which carries 48.45% of the original variability, is most affected by the data obtained from the release measurements at 1, 3, and 5 h. Component 2, which carries 27.87% of the original variability, is most affected by data obtained at measurement times of 5, 7, and 21 days. Thus, it can be said that while component 1 was most influenced by data obtained at the first phase of the release measurement, component 2 was most influenced by data obtained at the final phases of the measurement. Therefore, the PCA helped us to determine which phase of the release process contributed the most to the overall results obtained.

The PCA plot was used to graphically represent the differences between the samples with different compositions. Figure 6 shows that the samples form clearly distinguishable clusters depending on their different compositions. The liposome addition was evaluated using ANOVA, provided *p*-value = 0.0186 indicates statistically significant differences between different proteins; comparing samples with liposomes, the *p*-value = 0.0094 indicates that the differences between samples increased with the addition of liposomes.

The *p*-values for FGF2-STAB (*p* = 0.0095) and LYS (*p* = 0.0273) indicate that there is a statistically significant effect; however, in the case of HSA, the *p*-value (*p* = 0.4633) shows that the addition effect is not statistically significant. The statistically significant difference between FGF2-STAB and LYS and a statistically insignificant difference compared to HSA might result from the difference in isoelectric points (pI). While FGF2-STAB and LYS carry a net positive charge (pI = 9.6 in FGF2-STAB^60^ and 11 in LYS^50^) in physiological conditions, HSA exhibits a net negative charge of pI = 5^61^ and therefore might interact with the ITA groups on the polymer in quite a different manner.

The model proteins, HSA and LYS, vary in their total released amount and release mechanism. For HSA, rapid release was observed; approximately half of the incorporated protein was released within the first 3 days, and the release continued in the first-order kinetics as the concentration of HSA in the carrier gradually decreased in time.^62^ HSA is more of a hydrophobic protein bound in the core of the ABA-ITA micelles and has a considerable molecular weight (66.5 kg·mol^–1^). Due to the water uptake in the first 7 days and the molecular weight of the HSA, the HSA is eventually leaking from the aggregated micellar network. When HSA was encapsulated into liposomes, rapid release was observed only within the first day, as around 20% was released. Then, between days 1 and 5, no release was observed. After day 5, the second release step was recorded, until around 50% of the total HSA was released. In this case, the liposomes in a temperature-responsive hydrogel could function as an additional barrier; the drug encounters two barriers, resulting in a more sustained HSA release.^63^ Looking at LYS-containing matrixes, the total release of LYS is slightly improved in encapsulated formulations compared to that in unencapsulated LYS, changing the total released amount from 42 to 56%. Due to the strong affinity to the hydrophilic chains in the ABA-ITA micelles and low molecular weight, 40% of the encapsulated LYS was retained in the hydrogel even after 28 days.

The FGF2-STAB hydrogels exhibited a rapid release profile of the protein, with cumulative release in liposomal FGF2-STAB matrixes reaching 62% after 7 days. In contrast, the ABA-ITA matrix enriched with plain FGF2-STAB resulted in a release of 53% after 7 days, respectively. Between days 7 and 28, both matrixes provided a sustained release, with the liposomal FGF2-STAB matrix reaching 86% after 28 days, while the unencapsulated FGF2-STAB matrix resulted in a release of 67% after 28 days. The results correspond to the findings of Xu et al. on heparin-poloxamer encapsulated FGF2 release from the decellular spinal cord extracellular matrix. The findings on cumulative release correspond to the results on hydrolytic stability; as the FGF2-STAB interacts with the carboxyl ITA groups, the release is hindered to a certain level. The unencapsulated FGF2-STAB was released slowly which corresponds to the slower degradation of the enriched hydrogel, compared to the encapsulated FGF2-STAB in which case the degradation was much faster, which correlates with the faster release of the protein. Our results indicate that there is a major difference in the interactions comparing FGF2-STAB alone and encapsulated FGF2-STAB. Most probably, the liposomes do not tend to bind to the micellar structure as strongly as the protein alone. It seems that the interactions between ‘FGF2-STAB—hydrogel micelles’ are much stronger, and the protein is hindered to a certain level, holding the micellar network together, explaining the slower release and slower degradation. Compared to the ‘liposomal FGF2-STAB – hydrogel micelles’ interactions, in which case the liposomes present in between the micellar network rather tend to disrupt the stability of the micellar network from the beginning. This explains the faster degradation of the hydrogel and corresponds to the faster release of the protein as the protein cannot adhere to the micelles since they are “blocked” by the liposomes to a certain level. The liposomes have been proven as an effective carrier system for the delivery of low-molecular-weight proteins (FGF2-STAB and LYS) with a net positive charge, in which the protein release was enhanced, while in the case of large-molecular weight proteins (HSA) with a net negative charge, the total release mechanism was decreased.

### Conclusions

This study evaluated the influence of proteins and liposome-encapsulated proteins on the structural and hydrolytic stability of the itaconic acid (ITA)-modified PLGA-PEG-PLGA hydrogel. The liposomes used for this study were stable for 28 days with diameters below 100 nm with relatively significant encapsulation efficiencies. The system was proven effective for delivering human serum albumin-bonded drugs or for coencapsulation of drugs with lysozyme.

It was shown that proteins and liposome-encapsulated proteins do not impact the rheological properties of the itaconic acid-modified PLGA-PEG-PLGA hydrogel. The liposomes were proven to be an effective protective nanoparticle system for the delivery of the fibroblast growth factor-2 (FGF2-STAB) on its own; the main issue is that the isoelectric point of the FGF2-STAB (pI = 9.6) affected the hydrolytic stability of the ITA-modified PLGA-PEG-PLGA hydrogel. The combination of an FDA-approved, organic solvent-free hydrogel with “green” Mozafari method-prepared liposomes creates a unique eco-friendly system for the delivery of a commercially stable FGF2-STAB. This ITA-modified PLGA-PEG-PLGA system shows great promise for drug delivery applications. Additionally, these liposomes have been demonstrated to effectively enhance the release mechanism of FGF2-STAB, underscoring their potential in advanced therapeutic strategies. However, the study showed that the –COOH groups of ITA modification are affected by the net-positive charge of the protein leading to lower hydrolytic stability of micellar hydrogel, and therefore, in our further research, the carboxylic groups will have to be further blocked, replaced, or masked to reduce the matrix–protein interactions.

## Abbreviations

^1^H NMR: proton nuclear magnetic resonance
ABA-ITA: α,ω-itaconyl-poly(d,l-lactic acid-co-glycolic acid)-*b*-poly(ethylene glycol)-*b*-poly(d,l-lactic acid-co-glycolic acid)
AFG: autologous fat grafting
DLS: dynamic light scattering
DPPC: 1,2-dipalmitoyl-*sn*-glycerol-3-phosphocholine
EE: encapsulation efficiency
FDA: food and drug administration
FGF2-STAB: thermostable variant of fibroblast growth factor-2
GA: glycolic acid
GPC/SEC: gel permeation chromatography/size exclusion chromatography
HSA: human serum albumin
ITA: itaconic acid
LA: lactic acid
LIP: liposomal nanoparticles
LYS: human lysozyme
*M*_n_: number average molecular weight
PCA: principal component analysis
PDI: polydispersity index
PEG: poly(ethylene glycol)
PLGA-PEG-PLGA (ABA): poly(d,l-lactic acid-co-glycolic acid)-*b*-poly(ethylene glycol)-*b*-poly(d,l-lactic acid-co-glycolic acid)
ROP: ring opening polymerization
STEM: scanning transmission electron microscopy
T1: sol-to-gel transition, gelation temperature
T2: gel-to-sol transition, temperature of the beginning of the decay of the gel
THF: tetrahydrofuran
UPW: ultrapure water
UV–vis: ultraviolet–visible
ZP: zeta potential
